# Detection of retinal microvascular changes in von Hippel-Lindau disease using optical coherence tomography angiography

**DOI:** 10.1371/journal.pone.0229213

**Published:** 2020-02-20

**Authors:** Yifan Lu, Jay C. Wang, Rebecca Zeng, Tatsuo Nagata, Raviv Katz, Shizuo Mukai, John B. Miller

**Affiliations:** 1 Department of Ophthalmology, Harvard Medical School, Boston, MA, United States of America; 2 Harvard Retinal Imaging Lab, Massachusetts Eye and Ear, Boston, MA, United States of America; 3 Retina Service, Massachusetts Eye and Ear, Boston, MA, United States of America; University of Florida, UNITED STATES

## Abstract

**Purpose:**

Von Hippel-Lindau (VHL) disease is a hereditary disorder that can lead to ophthalmic manifestations, including retinal capillary hemangioma (RCH). The diagnosis of RCH is often guided by wide-field fluorescein angiography. In some cases, optical coherence tomography angiography (OCT-A) serves as a non-invasive alternative to FA. Herein, we used OCT-A to examine the macular microvasculature in patients with VHL disease.

**Subjects:**

Subjects were selected from patients with a diagnosis of VHL. The control group included eyes without retinal diagnosis from patients with an episode of unilateral retinal detachment or trauma and age ≤ 50 years old.

**Methods:**

Subjects were scanned on the Optovue RTVue-XR device to acquire 3mm x 3mm OCT-A images of the superficial (SCP) and deep capillary plexus (DCP). SCP and DCP vessel density (VD) were calculated after the images were binarized. Furthermore, for subjects with RCH, each OCT-A image was divided equally into four quadrants. SCP and DCP VD of quadrants with RCH were compared to those without RCH. T-tests were performed for statistical analysis.

**Results:**

67 eyes with a history of VHL disease were included as study subjects, while 16 eyes were included as controls. Significant increases in VD were found in patients with VHL disease for both the SCP (p = 0.0441) and DCP (p = 0.0344). When comparing quadrants with associated RCH development to those without, we found no significant difference in SCP VD (p = 0.160) or DCP VD (p = 0.484).

**Conclusions:**

OCT-A can detect changes in the retinal microvasculature in the macula of patients with VHL disease. OCT-A imaging may be an additional tool for screening and early detection of patients at risk of developing ocular complications of VHL disease. Future studies should explore subtle progression on OCT-A associated with the pathogenesis and development of RCH, particularly with larger scan patterns.

## Introduction

Von Hippel-Lindau (VHL) disease is a hereditary disorder caused by a germline mutation in the VHL tumor suppressor gene on chromosome 3p25.[1] Individuals carrying this mutation are often predisposed to developing benign and malignant tumors during childhood and adulthood. Common manifestations of VHL include cerebellar hemangioblastoma, renal cell carcinoma, pheochromocytoma, and pancreatic carcinoma.[2] The earliest and most common manifestation of VHL disease, however, is retinal capillary hemangioma (RCH), a benign vascular tumor of the retina.[3–5] Despite its benign nature, RCH can lead to severe clinical complications that affect vision such as retinal detachment, vitreous hemorrhage and neovascular glaucoma.[6]

RCH typically presents as a unilateral solitary tumor composed of proliferating capillaries and glial cells; however, bilateral and multifocal involvement can also be observed.[7–9] Most frequently, RCH occurs in the temporal peripheral retina, with involvement of the juxtapapillary region being a risk factor for poorer visual prognosis.[10] Current treatment modalities for RCH include cryotherapy,[11,12] laser photocoagulation,[13–15] photodynamic therapy,[16,17] vitreoretinal surgery,[18,19] brachytherapy,[20] and vitreoretinal surgery[21]. However, the treatment of RCH can be complicated by the development of new tumors.[22] It has been reported that permanent vision loss (vision < 20/400 in one or both eyes) can occur in up to 25% of cases even with adequate treatment.[23] Thus, early diagnosis, timely management, and routine follow-up are essential to the management of RCH in patients with VHL disease.

The diagnosis of RCH is based on examination findings and is most commonly guided by fluorescein angiography (FA) imaging, given the high degree of vascularity in VHL-related tumors. Additionally, B-scan, A-scan, and optical coherence tomography (OCT) of the macula can also be informative in aiding the diagnostic process.[24] Optical coherence tomography angiography (OCT-A), a novel imaging tool, has been increasingly utilized for evaluating the retinal microvasculature and detecting both retinal and choroidal diseases. Given its non-invasive nature and the ability to produce three-dimensional images, OCT-A is considered an adjunct to traditional imaging methods, such as FA.[25] Common clinical applications of OCT-A include the evaluation of diabetic retinopathy (DR), retinal vein occlusion (RVO), age-related macular degeneration (AMD), macular telangiectasia (MacTel) and other chorioretinal diseases.[26–30]

The current literature contains several case reports and clinical studies that offer potential applications of OCT-A in the screening and monitoring of RCH in VHL disease.[31–35] This retrospective study focuses on vessel density (VD) as a clinical parameter to study the use of OCT-A in detecting changes in the retinal microvasculature and development of ocular manifestations in patients with VHL disease.

## Methods

**Fig 1:**
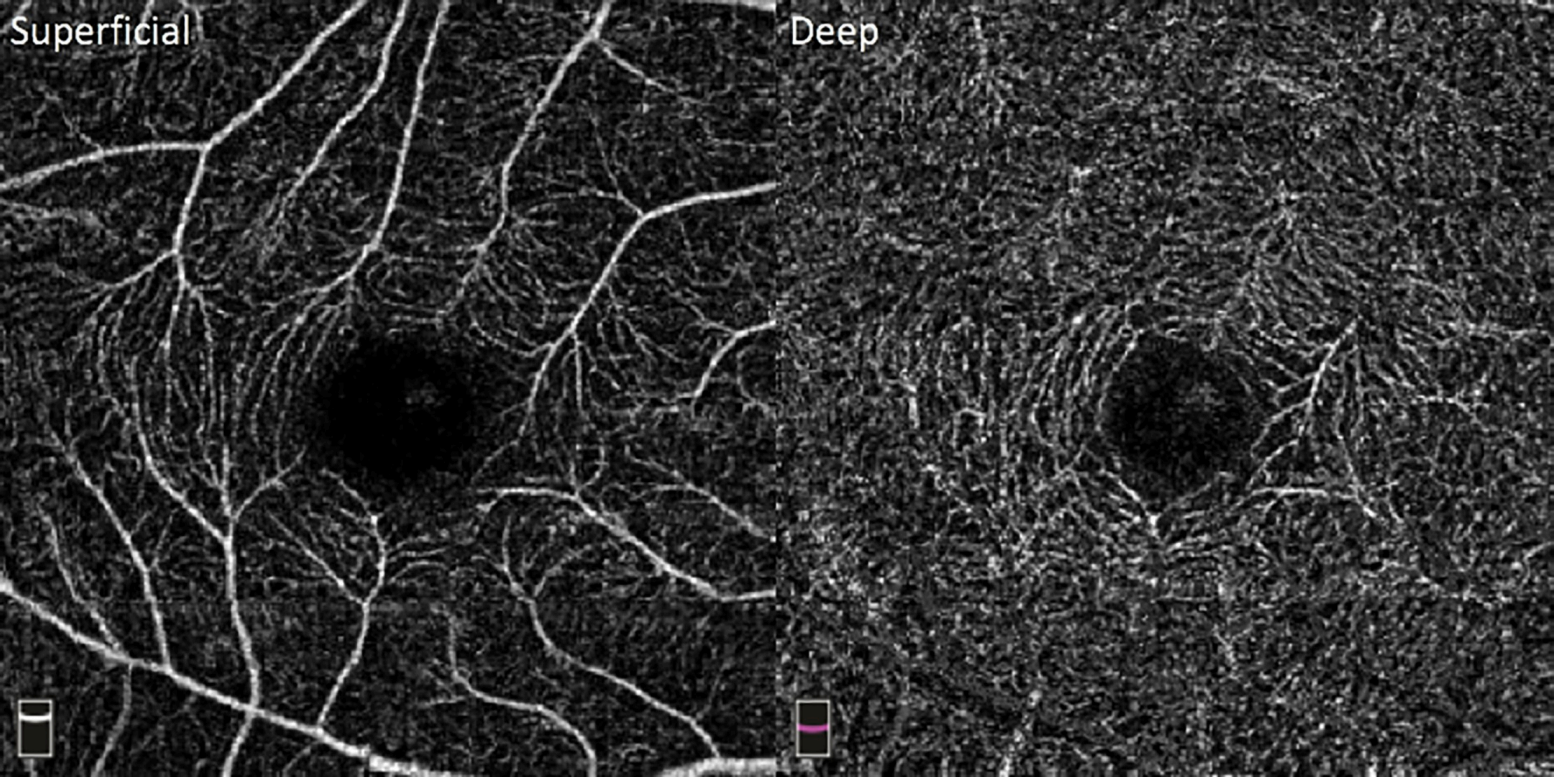
3mm x 3mm OCT-A imaging of the SCP and DCP using Optovue RTVue-XR.

Institutional Review Board (IRB)/Ethics Committee approval was obtained for this retrospective review. This study adhered to the tenets of the Declaration of Helsinki. An informed consent was obtained from each study subject. All OCT-A images were obtained using the Optovue RTVue-XR (Optovue Inc, Fremont, California, USA) device. This machine operates at 70,000 A-scans per second and employs an OCT-A algorithm of split-spectrum amplitude decorrelation angiography (SSADA). For each patient, pupillary dilation was completed and an OCT-A image with a scanning area of 3mm x 3mm centered on the macula was acquired. In addition, an image of the superficial capillary plexus (SCP) and deep capillary plexus (DCP) was generated for each OCT-A scan using preset layer segmentation (Fig 1).

The experimental group included patients from the Massachusetts Eye and Ear Infirmary (MEEI) retina clinics of SM and JBM. Inclusion was restricted to patients who had a diagnosis of VHL disease made with wide field FA, with or without RCH development. Patients with a history of other chorioretinal diseases, such as retinal detachment (RD), age-related macular degeneration (AMD), and diabetic retinopathy (DR), that could affect blood vessel density of the capillary plexuses were excluded from the study. The control group was selected from patients in the MEEI retina clinic with a history of a single episode of unilateral RD or unilateral traumatic eye injury. All the controls had no history of high myopia and were at age ≤ 50 years old. The uninvolved fellow eye of each patient was included in the control group. For each patient, 3mm x 3mm scans and images of the SCP and DCP were acquired from the Optovue RTVue-XR device.

**Fig 2:**
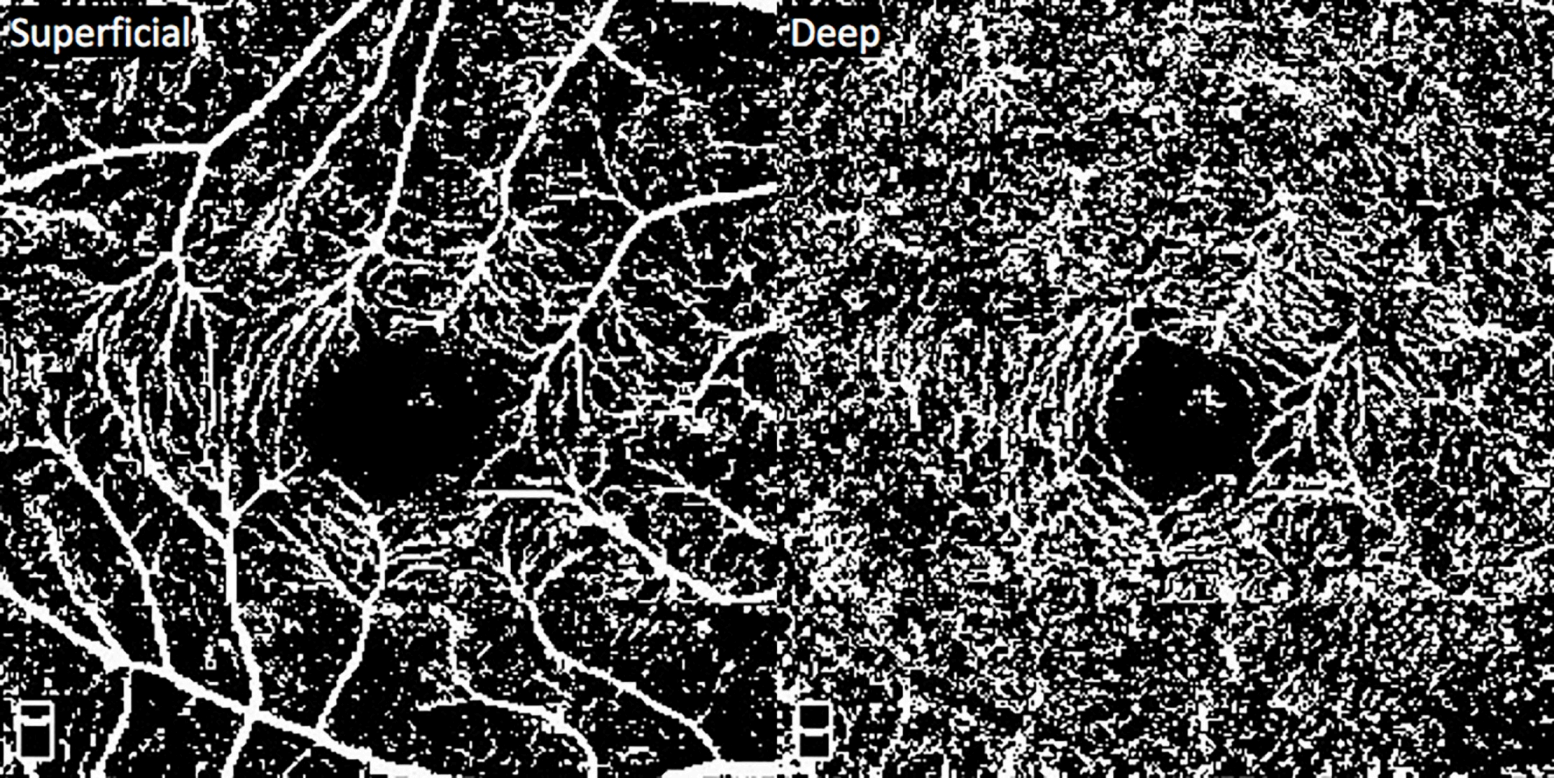
OCT-A imaging of the SCP and DCP after binarization using the Niblack automated local thresholding method.

**Fig 3:**
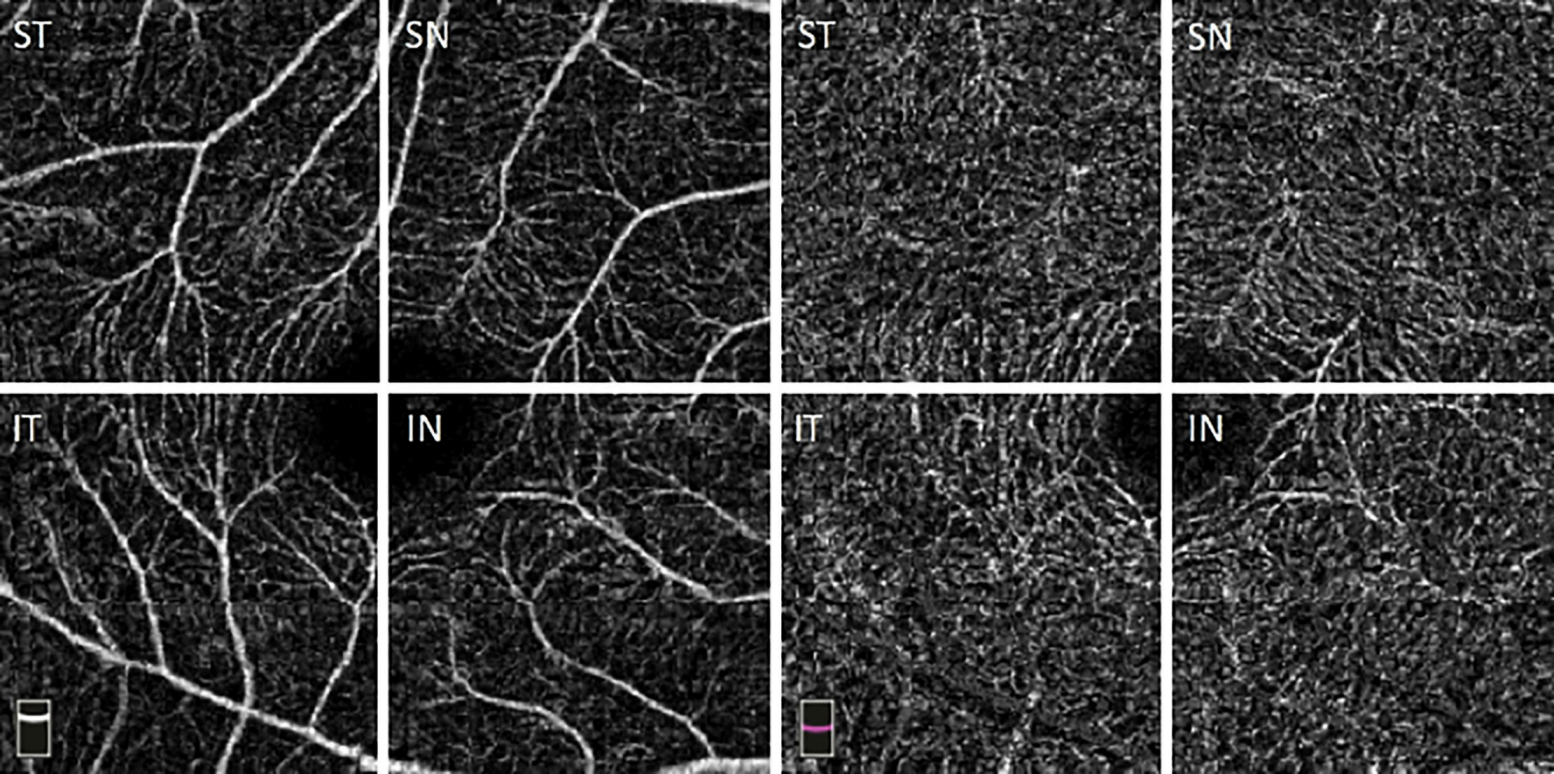
ST, SN, IT, IN quadrants of the 3mm x 3mm OCT-A imaging of the SCP and DCP.

Fiji software (National Institutes of Health, Maryland, USA) was utilized for image processing. VD calculations of the SCP and DCP were performed after the images were binarized using the Niblack automated local thresholding method (Fig 2). Furthermore, for study subjects with a RCH, each 3mm x 3mm OCT-A image was evenly divided into four quadrants to generate four images: the superotemporal quadrant (ST), the superonasal quadrant (SN), the inferotemporal quadrant (IT), and the inferonasal quadrant (IN) (Fig 3). For the image of each quadrant, VD was calculated after binarization.

Statistical analysis was performed using Microsoft Excel software (Microsoft Corporation, Redmond, WA). Two-tailed t-tests were performed to analyze for differences in VD between patients with VHL and patients in the control group, between VHL patients with history of RCH and no RCH, and between VHL patients without history of RCH and patients in the control group. An additional two-tailed t-test was conducted to compare the VD of quadrants with a history of RCH development to those without a history of RCH development. A p-value of less than 0.05 was considered to be significant.

## Results

In this study, 67 eyes from 39 patients with a history of VHL disease were included (Table 1). The mean age of patients with VHL disease was 29 ± 15 years old, while the gender distribution was 24 females (62%) and 15 males (38%). The control group included 16 eyes from 16 patients without a history of VHL disease. The mean age of the control group was 35 ± 12 years old, while the gender distribution was 6 females (38%) and 10 males (62%).

**Table 1:** Demographic information and RCH development in patients with VHL disease.

Patient	Gender	Eye	Quadrants with RCH (OD)				Quadrants with RCH (OS)			
			ST	SN	IT	IN	ST	SN	IT	IN
1	F	OU								
2	F	OU						Y	Y	
3	M	OU	Y							
4	M	OS								
5	F	OU							Y	
6	M	OU	Y		Y					
7	F	OD								
8	M	OU			Y				Y	
9	M	OU					Y		Y	
10	F	OU					Y			
11	F	OU			Y		Y			
12	M	OU	Y				Y			
13	F	OU			Y					
14	F	OD								
15	F	OU							Y	
16	M	OU				Y				
17	F	OS								
18	M	OS								
19	F	OU								
20	M	OU								
21	M	OD								
22	F	OU							Y	
23	F	OS								
24	F	OU								
25	F	OU	Y							
26	M	OU			Y					
27	M	OU								
28	M	OD								
29	F	OD		Y	Y					
30	F	OS								
31	M	OU				y			Y	
32	F	OS					Y	Y	Y	
33	F	OU		Y					Y	
34	F	OU								
35	M	OU								
36	F	OU								
37	F	OU		Y						
38	F	OU					Y		Y	Y
39	F	OU	Y					Y		

OU = both eyes, OD = right eye, OS = left eye, ST = superotemporal quadrant, SN = superonasal quadrant

IT = inferotemporal quadrant, IN = inferonasal quadrant, Y = yes

For SCP, there was a statistically significant greater mean VD of VHL patients 0.335 ± 0.00014 compared to controls 0.328 ± 0.00010 (p = 0.0441). Similarly, the DCP also demonstrated a statistically significant increase greater VD of patients with VHL 0.369 ± 0.00027 compared to controls 0.360 ± 0.00017 (p = 0.0344) (Table 2).

**Table 2:** SCP VD and DCP VD comparisons between patients with and without VHL disease.

* *	Mean	SD	p value
SCP VD VHL	0.335	0.00014	0.0441
SCP VD Control	0.328	0.00010	
DCP VD_VHL	0.369	0.00027	0.0344
DCP VD_Control	0.360	0.00017	

Of the 67 eyes included in the VHL study group, 28 eyes had a history of RCH treated by laser ablation, and 39 eyes had never shown RCH. Interestingly, the eyes with RCH treated with laser demonstrated reductions in VD compared to those without RCH. While this trend did not reach statistical significance for the SCP, there was a statistically significant reduction in the DCP, with VD of laser treated RCH eyes of 0.363 ± 0.00036 compared to 0.374 ± 0.00015 in those eyes without RCH (p = 0.008) (Table 3).

**Table 3:** SCP VD and DCP VD comparisons between eyes with and without history of RCH development.

* *	Mean	SD	p value
SCP VD RCH	0.332	0.00018	0.168
SCP VD No RCH	0.336	0.00011	
DCP VD RCH	0.363	0.00036	0.008
DCP VD No RCH	0.374	0.00015	

When comparing these subgroups to controls, only VHL eyes without previous RCH development demonstrated significantly higher VD for both the SCP (p = 0.0138) and DCP (p = 0.0018). There were no significant differences identified when comparing laser treated RCH eyes to controls at either SCP (p = 0.309) or DCP (p = 0.691) (Tables 4 and 5).

**Table 4:** SCP VD and DCP VD comparisons between VHL eyes with history of RCH and control eyes without VHL disease.

* *	Mean	SD	p value
SCP VD RCH	0.332	0.00018	0.309
SCP VD Control	0.328	0.00010	
DCP VD_RCH	0.363	0.00036	0.691
DCP VD Control	0.360	0.00017	

**Table 5:** SCP VD and DCP VD comparisons between VHL eyes without history of RCH and control eyes without VHL disease.

* *	Mean	SD	p value
SCP VD No RCH	0.336	0.00011	0.0138
SCP VD Control	0.328	0.00010	
DCP VD No RCH	0.374	0.00015	0.0018
DCP VD Control	0.360	0.00017	

Among the 28 eyes with a diagnosis of RCH, 36 quadrants were identified with history of RCH treated by laser ablation. The most common location of RCH was the IT quadrant (n = 16, 44%), followed by the ST (n = 11, 31%), SN (n = 6, 17%), and IN (n = 3, 8%) quadrants. No significant difference was detected between quadrants with laser treated RCH and quadrants without RCH development in either SCP VD (0.336 ± 0.00021 vs. 0.331 ± 0.00022, p = 0.160) or DCP VD (0.365 ± 0.00049 vs. 0.362 ± 0.00037, p = 0.484) (Table 6).

**Table 6:** SCP VD and DCP VD comparisons between quadrants with laser treated RCH and quadrants with no history of RCH.

* *	Mean	SD	p value
SCP VD Q w/ RCH	0.336	0.00021	0.160
SCP VD Q w/t RCH	0.331	0.00022	
DCP VD Q w/ RCH	0.365	0.00049	0.484
DCP VD Q w/t RCH	0.362	0.00037	

Q w/ RCH = quadrants with history of RCH treated by laser ablation, Q w/t RCH = quadrants with no history of RCH development.

## Discussion

VHL disease is a genetic disorder that leads to cancer development in various tissues and organs in the body, such as the kidneys, brain, pancreas, and retina.[36] RCH, the earliest and most common manifestation of VHL disease, can cause serious vision-threatening complications, even with proper treatment due to the development of new tumors in different sites of the retina.[22] Thus, the ability to detect small RCH tumors through early screening and monitoring is critical to the management of patients with VHL disease. This retrospective review demonstrates the ability of OCT-A to identify retinal microvasculature changes in the macula of patients with VHL compared to controls. This study may indicate a novel approach with OCT-A to evaluate for potential ocular complications of VHL disease. Improved risk stratification through continued investigation of this technique may improve screening recommendations and clinical prognosis.

The present study focused on VD of the macula as the primary metric for comparison, given the ease of acquisition and its reliability as an indicator of retinal disease activity.[37] Our results showed that VD in the SCP and DCP layers was significantly greater in VHL eyes than in eyes without VHL disease, specifically in VHL eyes without history of RCH development. These findings are consistent with the pathogenesis of tumor development in VHL, wherein an accumulation of hypoxia-inducible factor (HIF), secondary to a lack of degradation by VHL protein, induces the expression of vascular endothelial growth factor (VEGF).[38,39] Similar vascular changes driven by up-regulation of HIF and VEGF are also seen in other systemic manifestations of VHL disease, such as renal cell carcinoma.[40,41] The increase in the macular VD metric is of clinical interest given the potential for additional study of OCT-A imaging as a quick and non-invasive screening tool for the ocular manifestations of VHL.

An interesting finding in this study is that although an increased VD was observed in VHL eyes compared to control eyes, this increase was only found in VHL eyes without RCH development. Contrarily, the eyes with a history of RCH treated by laser ablation demonstrated reductions in VD in comparison to VHL eyes without RCH. This finding could be a result of the fact that laser ablation therapy destroys the blood flow to RCH and thus lowers the macular VD. A case report studying the OCT-A findings of a retinal hemangioblastoma pre- and post-laser therapy found a remarkable constriction of vessels associated with the hemangioblastoma and a significantly reduced blood flow to the tumor after laser ablation therapy.[42] In another case report, the OCT-A scan of a retinal racemose hemangioma with retinal artery microaneurysm showed a reduction in flow signal and a decrease in blood flow to the lesion after treatment with focal laser therapy.[43] These findings suggest that a prospective study with OCT-A imaging on RCH eyes without previous laser ablation may give further insight into the application OCT-A in monitoring the development of RCH in VHL patients.

RCH lesions are most commonly observed by dilated fundus examination. Additional RCH lesions can be found with wide field FA. However, FA requires a skilled healthcare provider and photographer, and carries the risk of allergic reaction. We wondered if there might be regional changes in the retinal microvasculature that could help raise suspicion for peripheral RCH lesions. In order to do so, we looked for differences in VD between quadrants with RCH development compared to quadrants without a history of RCH. Unfortunately, our results did not show a significant difference for either SCP VD (p = 0.160) or DCP VD (p = 0.484). This may in part be due to the smaller testing region as we only used 3x3 mm macular scans. Though we found no significant difference in this study, additional work with larger scanning patterns may provide more helpful biomarkers of disease activity.

There are limitations that should be given consideration in this study. A major limitation is that the control group has a relatively small size given the strict exclusion criteria of healthy eyes and the age limitation of ≤ 50 years old to prevent age difference from being a confounding factor. Another limitation of this study is that some of the OCT-A images were not precisely centered on the macula; thus, the macular vessels were not evenly distributed when the images were divided into quadrants, which could potentially affect the VD measurement. Therefore, a prospective study with larger sample size and more precise imaging is indicated.

In conclusion, OCT-A has the ability to detect changes in retinal microvasculature densities in patients with VHL disease. Given this finding, OCT-A imaging should be considered an adjunctive method for the screening and early detection of VHL disease in patients at risk of developing ocular manifestations. Future studies should focus on the clinical applications of OCT-A in monitoring for the development of RCH, especially in detecting subtle changes in VD in specific quadrants with larger scan patterns.

## Supporting information

S1 Data (XLSX)
